# Single-Shot Multi-Frequency 3D Shape Measurement for Discontinuous Surface Object Based on Deep Learning

**DOI:** 10.3390/mi14020328

**Published:** 2023-01-27

**Authors:** Min Xu, Yu Zhang, Yingying Wan, Lin Luo, Jianping Peng

**Affiliations:** School of Physical Science and Technology, Southwest Jiaotong University, Chengdu 610031, China

**Keywords:** fringe projection profilometry, deep learning, phase retrieval

## Abstract

It is challenging to stably and rapidly achieve accurate absolute phase retrieval for isolated objects with a single-shot pattern in fringe projection profilometry (FPP). In this context, a single-shot multi-frequency absolute phase retrieval (SAPR-DL) method based on deep learning is proposed, which only needs to capture one fringe image to obtain the full-field precise absolute phase. Specifically, a low-frequency deformed fringe image is loaded into the trained one-to-two deep learning framework (DLFT) to predict unit-frequency and high-frequency deformed fringe images. Then, three fringe images with different frequencies are loaded into the trained deep learning phase retrieval framework (DLPR) to calculate the corresponding absolute phase. The experimental results prove that the proposed SAPR-DL method can obtain the three-dimensional (3D) shape measurement of multiple complex objects by collecting a single-shot fringe image, showing great prospects in advancing scientific and engineering applications.

## 1. Introduction

As an important research direction in the field of computer vision, 3D reconstruction technology plays an important role which has extensive application value. Common 3D reconstruction techniques include multi-view stereo [1], time of flight [2], structured light [3] and shape from shading and photometric stereo [4,5,6]. The structured light method is widely used in the actual visual measurement system because of its simple calculation, high measurement accuracy and non-contact characteristics. Fringe projection profilometry (FPP) has become one of the most promising optical 3D measurement techniques due to its simple hardware structure, flexible implementation and high precision. To achieve 3D measurement in high-speed scenarios, it is usually necessary to improve measurement efficiency by reducing the number of images required for each measurement. The most ideal way is to obtain 3D data from a single-shot image. The Fourier transform (FT) method [7,8,9,10] is the most popular single-shot fringe analysis method which uses a band-pass filter to extract the phase information contained in the frequency domain. Unfortunately, spectral aliasing can introduce large phase errors in discontinuous and isolated regions. Hence, researchers have designed a variety of special fringe composite methods [11,12] by spatial frequency reuse strategies [13,14,15], expecting to achieve high-precision 3D measurement. However, spectral aliasing still occurs, which leads to lower phase quality around discontinuities and isolated regions. Therefore, these methods are not applicable to high-precision 3D measurement.

In recent years, many researchers have applied deep learning methods to high-precision phase retrieval of single-shot fringe images; however, only wrapped phase is obtained [16,17,18]. The wrapped phase needs to be unwrapped before 3D shape measurement. The most commonly used phase unwrapping method is the temporal phase unwrapping (TPU) algorithm [19], which requires multiple fringe patterns to obtain the absolute phase but with lower measurement efficiency. Furthermore, some researchers have combined deep learning with composite fringe projection, expecting higher accuracy and more robust phase retrieval and 3D reconstruction from a single composite fringe pattern. Li et al. proposed composite fringe projection deep learning profilometry [20,21], reconstructed the 3D shape information of target objects by constructing a convolutional neural network and analyzed single-shot spatial frequency multiplexed fringe patterns. However, a proper composite fringe image needs to be designed in advance. Qian et al. proposed a single-shot geometry constraint and phase unwrapping method based on deep learning [22] which can meet the requirements of single-shot projection but requires two cameras, thus leading to higher hardware cost. Moreover, researchers established the mapping relationship between fringe image and absolute depth information by an end-to-end approach and extracted 3D information of objects from a single-shot image [23,24,25]. Unfortunately, it is difficult for the proposed method to obtain high-precision measurement results in practical applications due to its inability to avoid fringe ambiguity.

In this paper, we propose a single-shot multi-frequency absolute phase retrieval (SAPR-DL) method based on deep learning. A one-to-two deep learning framework (DLFT) is designed and trained to convert one low-frequency fringe image into one unit-frequency fringe image and one high-frequency fringe image. Furthermore, we design and train another deep learning phase retrieval framework (DLPR) to achieve the absolute phase retrieval of objects from three fringe images (unit-frequency, low-frequency and high-frequency). Our DLPR network replaces the cumbersome steps involved in the traditional phase retrieval method, such as wrapped phase extraction and phase unwrapping. Only one fringe image needs to be captured to achieve high-quality absolute phase retrieval of discontinuous and isolated objects.

## 2. Methods

### 2.1. Principle of Single-Shot Multi-Frequency Absolute Phase Retrieval Method Based on Deep Learning

The ultimate goal of FPP technology is to retrieve 3D data from a single-shot image. Inspired by the recent successes of deep learning technology in FPP, we consider obtaining the absolute phase from a single-shot fringe image by deep neural networks. However, the reliability of deep learning largely depends on the original input information, and deep learning network is not always reliable in the case of ambiguous input. Therefore, in order to eliminate the phase singularity robustly, it is of great importance to design an explicit input pattern. Specifically referring to the traditional temporal phase unwrapping (TPU) algorithm, three groups of phase-shifted fringe patterns with different frequencies are projected. Among them, three fringe patterns with different frequencies are selected as the input of the DLPR network, and the absolute phase calculated by the traditional TPU algorithm is used as the ground truth for training the DLPR network. The three deformed fringe images collected by CCD can be expressed as:

(1) $$I_{u}^{c}\left(x^{c},y^{c}\right)=A\left(x^{c},y^{c}\right)+B\left(x^{c},y^{c}\right)\cos\left(2\pi f_{u}^{c}x^{c}\right)$$

(2) $$I_{l}^{c}\left(x^{c},y^{c}\right)=A\left(x^{c},y^{c}\right)+B\left(x^{c},y^{c}\right)\cos\left(2\pi f_{l}^{c}x^{c}\right)$$

(3) $$I_{h}^{c}\left(x^{c},y^{c}\right)=A\left(x^{c},y^{c}\right)+B\left(x^{c},y^{c}\right)\cos\left(2\pi f_{h}^{c}x^{c}\right)$$

where $\left(x^{c},y^{c}\right)$ are the coordinates of captured image; $A\left(x^{c},y^{c}\right)$ is average intensity; $B\left(x^{c},y^{c}\right)$ is the intensity modulation. $f_{u}^{c}$, $f_{l}^{c}$, $f_{h}^{c}$ are the carrier frequencies of unit-frequency, low-frequency and high-frequency fringe images, respectively.

**Figure 1:**
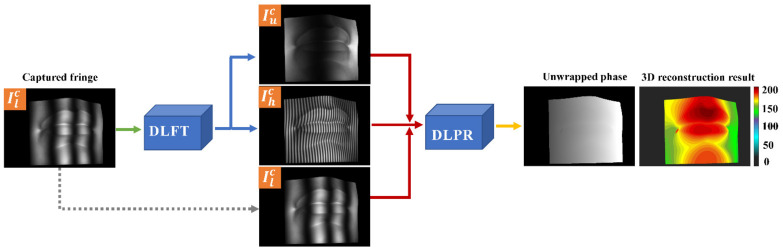
The implementation process of the proposed SAPR-DL method.

We combined three fringe images of different frequencies to calculate the absolute phase through the DLPR network. To achieve the goal of retrieving 3D data from single-shot image, we designed a DLFT network model. The DLFT network achieved one-to-two fringe images transformation and converted the captured low-frequency fringe images into unit-frequency and high-frequency ones to ensure that the inputs of the DLPR network are not ambiguous. The acquired low-frequency fringe is used as the input for training the DLFT network, and unit-frequency and high-frequency fringes are used as the ground truth for training the DLFT network. Once the DLFT model is trained, it is only necessary to input one low-frequency fringe image into the trained DLFT model to obtain the other two fringe images of different frequencies. It is worth mentioning that the output data of the DLFT model also serve as the input data of the DFPR model. To describe the implementation steps of this method more concisely in the following text, we named it the SAPR-DL method. The implementation process of SAPR-DL method is shown in Figure 1. In Section 2.2, we will describe in detail the data set construction process required for training DLFT and DLPR models.

### 2.2. Training Data Set

Data-driven deep learning methods use a large quantity of training data to train the model so that its predicted output can be infinitely close to ground truth. To train the DLFT model, we need to collect three kinds of frequency fringe images to build corresponding data sets, among which deformed unit-frequency and high-frequency fringe images are used as the ground truths of the DLFT network, and low-frequency ones are used as the input data of the DLFT network. In addition, we also need high-quality and unambiguous absolute phase (ground truth) of the object to train the DLPR network.

To be specific, we used N-step phase-shift profilometry (PSP) for wrapped phase retrieval. As we all know, PSP can achieve high-precision pixel-by-pixel phase measurement without being affected by ambient light. In this paper, standard N-step phase-shift fringe patterns are used, and the intensity of projected fringe patterns can be expressed as:

(4) $$I_{n}^{p}\left(x^{p},y^{p}\right)=a^{p}+b^{p}\cos\left(2\pi f^{p}x^{p}-2\pi n/N\right),\;n=0,1,\ldots N-1$$

where $I_{n}^{p}\left(x^{p},y^{p}\right)$ is the fringe patterns to be projected; $\left(x^{p},y^{p}\right)$ are the coordinates of projector pixel. n is the phase shift index; $a^{p}$ is background; $b^{p}$ is amplitude. $f^{p}$ is fringe frequency; $f^{p}=\frac{F}{W}$, F is the total number of fringe periods; W is the horizontal resolution of the projection pattern. These fringe patterns are sequentially projected onto the object surface, and deformed fringe patterns captured by the camera can be expressed as:

(5) $$I_{n}^{c}\left(x^{c},y^{c}\right)=A\left(x^{c},y^{c}\right)+B\left(x^{c},y^{c}\right)\cos\left[\varphi \left(x^{c},y^{c}\right)-2\pi n/N\right],\;n=0,1,\ldots N-1$$

where $\varphi \left(x^{c},y^{c}\right)$ is the phase distribution of the object to be measured. According to the least square algorithm, the wrapped phase $\varphi \left(x^{c},y^{c}\right)$ can be obtained as [26]:

(6) $$\varphi \left(x^{c},y^{c}\right)=\tan^{-1}\frac{\sum_{0}^{N-1}I_{n}^{c}\left(x^{c},y^{c}\right)\sin(2\pi n/N)}{\sum_{0}^{N-1}I_{n}^{c}\left(x^{c},y^{c}\right)\cos(2\pi n/N)}$$

To obtain high-precision phase distribution, the standard 16 step phase-shift fringe patterns with an offset of 2*π*/16 are used; that is, *N* = 16. Due to the truncation property of arctan function, the obtained phase $\varphi \left(x^{c},y^{c}\right)$ is wrapped in the (−*π*, *π*]. The relationship between $\varphi \left(x^{c},y^{c}\right)$ and absolute phase $\Phi \left(x^{c},y^{c}\right)$ is expressed as:

(7) $$\Phi \left(x^{c},y^{c}\right)=\varphi \left(x^{c},y^{c}\right)+2\pi k\left(x^{c},y^{c}\right)$$

where $\Phi \left(x^{c},y^{c}\right)$ is the absolute phase; $\varphi \left(x^{c},y^{c}\right)$ is the wrapped phase calculated from Equation (6); $k\left(x^{c},y^{c}\right)$ is the integer phase order. To obtain continuous phase distribution, we use the multi-frequency temporary phase unwrapping (MF-TPU) algorithm [27] to unwrap the wrapped phase $\varphi \left(x^{c},y^{c}\right)$. The multi-frequency temporary phase unwrapping algorithm uses two additional phase maps of different fringe frequencies to unwrap the phase. $\varphi_{u}\left(x^{c},y^{c}\right)$, $\varphi_{l}\left(x^{c},y^{c}\right)$ and $\varphi_{h}\left(x^{c},y^{c}\right)$ are wrapped phases corresponding to the unit-frequency, low-frequency and high-frequency, respectively. $k_{l}\left(x^{c},y^{c}\right)$ and $k_{h}\left(x^{c},y^{c}\right)$ represent the fringe orders of the low-frequency and high-frequency. They can be determined as:

(8) $$\left\{\begin{array}{l} k_{l}\left(x^{c}{,y}^{c}\right)=\mathrm{Round}\;\left[\frac{\left(T_{l}{/T}_{u}\right)\Phi_{u}\left(x^{c}{,y}^{c}\right){-\varphi }_{l}\left(x^{c}{,y}^{c}\right)}{2\pi }\right]\\ k_{h}\left(x^{c}{,y}^{c}\right)=\mathrm{Round}\;\left[\frac{\left(T_{h}{/T}_{l}\right)\Phi_{l}\left(x^{c}{,y}^{c}\right){-\varphi }_{h}\left(x^{c}{,y}^{c}\right)}{2\pi }\right] \end{array}\right.$$

where Round [] is the rounding function to obtain the nearest integer value. The total number of the periods of the fringes are $T_{u}$, $T_{l}$ and $T_{h}$ for the unit-frequency, low-frequency and high-frequency fringe patterns, respectively. $\Phi_{u}\left(x^{c},y^{c}\right)$ is the unwrapped phase of the unit-frequency fringe image. $\Phi_{l}\left(x^{c},y^{c}\right)$ is the absolute phase of low-frequency fringe image. The absolute phase of high-frequency fringe image $\Phi_{h}\left(x^{c},y^{c}\right)$ can be obtained using Equation (7) and is used for the final 3D shape reconstruction. In this paper, the total numbers of projected fringe periods are $T_{u}$ = 1, $T_{l}$ = 6 and $T_{h}$ = 50, respectively.

To obtain enough data for network training, we constructed a monocular-structured light lighting system consisting of a projector (model: TI LightCrafter4500) and an 8-bit industrial camera (model: acA1920-40gm, Basler) with a lens with a focal length of 8 mm. The projector has a resolution of 912 × 1140 and a projection pattern rate of 120 Hz with 8-bit. To make the system as small as possible, the angle between the projector and the camera is set to 15°, and the distance between object and the imaging system is set to about 30 cm. The horizontal resolution of projector is W = 912; the total numbers of projected fringe periods are $T_{u}$ = 1, $T_{l}$ = 6 and $T_{h}$ = 50; the fringe periods are $F_{u}$ = W/$T_{u}$, $F_{l}$ = W/$T_{l}$ and $F_{h}$ = W/$T_{h}$, respectively. In our experiment, we collected the data including 680 different gypsum models scenarios, where 20 simple and complex objects were randomly combined.

### 2.3. Network Architecture

**Figure 2:**
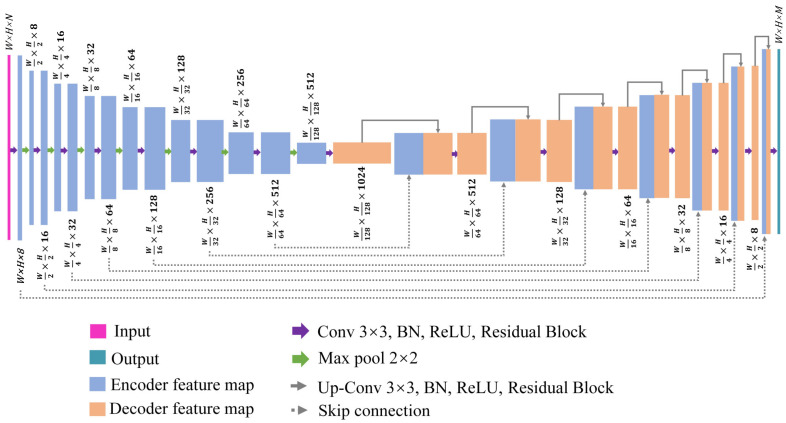
Architecture of the designed DLFT/DLPR network.

**Figure 3:**
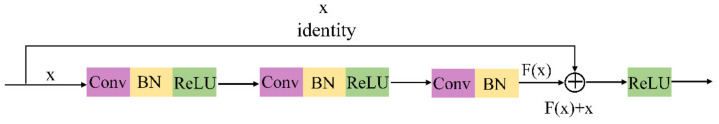
Architecture of the residual structure. The x represents the input data.

The structure of DLFT/DLPR network is illustrated in Figure 2. As can be seen from the figure, the DLFT/DLPR network consists of a U-shaped structure [28] and a residual structure [29]. The left part of the U-shaped structure uses the encoder structure of repeated convolution + down-sampling to extract features, and the right part uses the decoder structure of repeated deconvolution + up-sampling to reconstruct images. As far as the encoder path is concerned, each encoder layer consists of the following operations: two 3×3 convolutions, batch normalization (BN) [30] and rectified linear unit (ReLU) [31], a residual structure remaining blocks and max pooling. In addition, each decoder layer in the decoder path includes up-sampling, cascading with corresponding clipped feature mapping in the encoder path via skip connection, two 3 × 3 convolutions (followed by BN and ReLU) and the residual block between the two convolutions. The residual structure is shown in Figure 3. The residual structure establishes a shortcut between input and output to avoid the disappearance of gradient caused by increased depth in the deep neural network, thus ensuring the local details of the output results. The input layer of the DLFT is high-frequency fringe image, and the output layer are one unit-frequency fringe image and one low-frequency fringe image, that is, *N* = 1, *M* = 2. The input layer of the DLPR network contains unit-frequency, low-frequency and high-frequency fringe images, and the output layer is one absolute phase map; that is, *N* = 3, *M* = 1.

### 2.4. Network Training

Our DLPR and DLFT networks were trained on the PyTorch platform using a workstation equipped with a Core Intel(R) Xeon(R) Silver 4214R CPU (2.4 GHz), 32 GB of RAM and 1 × NVIDIA GeForceGTX-3090Ti GPU. We randomly divided the data into 80% (544), 10% (68) and 10% (68) as training, validation and test data sets, respectively. In addition, the image size was clipped to 640 × 896 px^2^ in order to successfully input the captured image into the network. The DLPR network employed an Adam optimization [32] with a learning rate of 0.001, which was reduced to 10% after every 300 iterations. The SSIM + Sobel loss, a loss metric proposed by Ref. [33], was used as the cost function in the experiment. MAE (L1) loss function was adopted in DLFT network, which was different from the DLPR network. Our DLFT network employed an Adam optimization with a learning rate of 0.001, which was reduced to 10% after every 200 iterations. In the training process, Xavier initialization [34] was adopted to initialize the network weights. After iterations of no more than 1500 epochs, we obtained the optimal model. More importantly, fringe images were normalized before being loaded into the network to simplify model learning. All the objects used in the testing process did not appear in the training stage. The CUDNN library [35] was used to optimize parallel execution in the whole training process.

## 3. Experiments and Results

### 3.1. Generation of Fringe Images by DLFT Network

To verify the performance of the proposed SAPR-DL method, we tested four scenarios that had never been seen in the network. These scenarios include a single object with a continuous surface and a combination of multiple objects with isolated surfaces. We conducted a series of contrast experiments to explain why the low-frequency fringe image was selected as the input of DLFT network instead of unit-frequency or high-frequency fringe images. To more accurately quantify the difference between the fringe image generated by the DLFT network and the ground truth, we calculated the structure similarity (SSIM) [36], an index to measure the similarity between two images, which is a number between 0 and 1. The closer the SSIM index is to 1, the higher the similarity between the SSIM index and the corresponding ground truth. The SSIM is calculated by

(9) $$SSIM(I,G)=\frac{\left(2\mu_{I}\mu_{G}+c_{1}\right)\left(2\sigma_{IG}+c_{2}\right)}{\left(\mu_{I}^{2}+\mu_{G}^{2}+c_{1}\right)\left(\sigma_{I}^{2}+\sigma_{G}^{2}+c_{2}\right)}$$

where I is the fringe image generated by the DLFT network, and G is the ground truth. $\mu_{I}$ is the mean of the image I; $\mu_{G}$ is the mean of the ground truth G; $\sigma_{I}$ and $\sigma_{G}$ are the variances of I and G, respectively. $\sigma_{IG}$ is the covariance of I and G, and $c_{1}$, $c_{2}$ are two constants used to stabilize the calculation.

The fringe images with different frequencies were input into the DLFT network in turn. The SSIM index of the other two fringe images generated are shown in Table 1. It can be seen from Table 1 that the SSIM index output by the network was relatively low when the fringe image of unit-frequency was used as the input of the DLFT network. For example, the SSIM index of the high-frequency fringe image generated in scenario 3 was only 0.932. The SSIM index of the generated image was greater than 0.950, whether a low-frequency or high-frequency fringe image was used as the input of DLFT network. However, the SSIM index of the fringe images generated in three different cases is highly similar. In order to select the input fringe image reasonably, we use the trained DLPR model to test the above three different cases on the whole test set, with phase errors of 0.68 rad, 0.20 rad and 1.38 rad, respectively. We find that the phase error is the smallest when the low-frequency fringe image is used as the input of DLFT network. The possible reason for this is that maintaining the integrity of a low-frequency fringe image is more important in phase calculation. In the later experiment, we use the low-frequency fringe image as the input of DLFT network.

**Figure 4:**
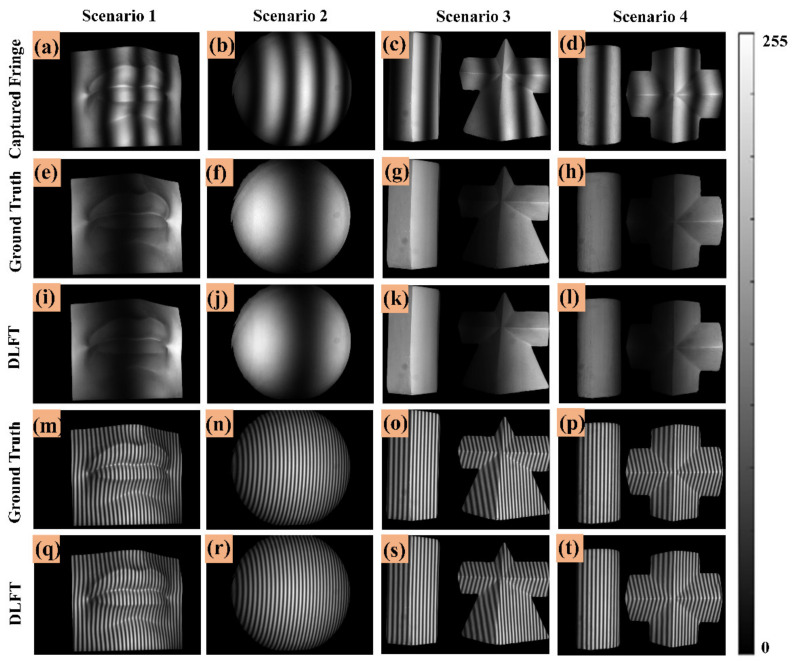
Fringe images generation result based on DLFT network. (**a**–**d**) Captured low-frequency fringe images. (**e**–**h**) Ground truths (unit-frequency). (**i**–**l**) Unit-frequency fringe images generated by DLFT network. (**m**–**p**) Ground truths (high-frequency). (**q**–**t**) High-frequency fringe images generated by DLFT network.

To more intuitively illustrate the performance of fringe images generated by DLFT network, the fringe transformation results of the above four scenarios are shown in Figure 4. Figure 4a–d are the inputs of DLFT network, which are low-frequency fringe images. Figure 4e–h are the unit frequency fringe images as ground truths, and Figure 4i–l are the unit-frequency fringe images generated by DLFT network. Figure 4m–p are high-frequency fringe images as ground truths, and Figure 4q–t are high-frequency fringe images generated by DLFT network. According to the above results, the unit-frequency and high-frequency fringe images generated by DLFT network were very similar to their corresponding ground truths. The experimental results indicated that only one low-frequency fringe image needed to be captured to generate one unit-frequency fringe image and one high-frequency fringe image through DLFT network. Based on the above method, the multi-frequency fringe information required for absolute phase calculation can be supplemented.

### 3.2. Extracting Absolute Phase by DLPR Network

**Figure 5:**
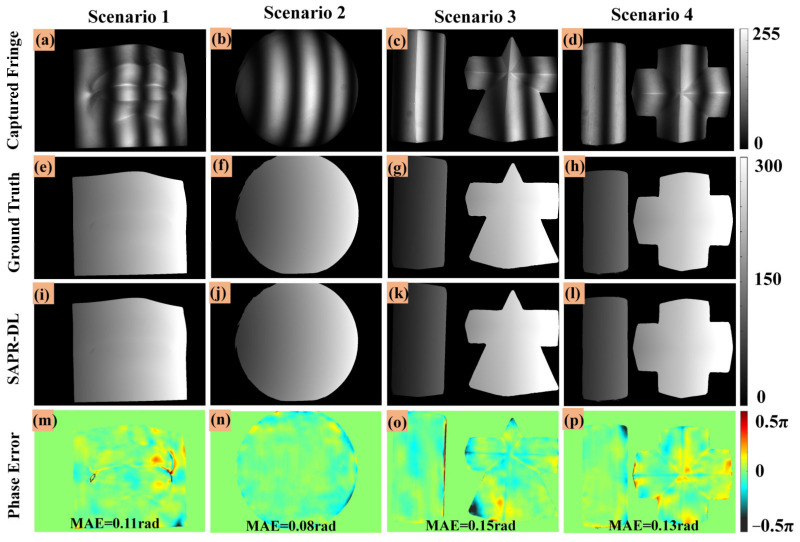
Absolute phase retrieval results based on the proposed SAPR-DL method. (**a**–**d**) The captured low-frequency fringe images. (**e**–**h**) Absolute phase maps (ground truths). (**i**–**l**) Absolute phase maps (SAPR-DL). (**m**–**p**) The absolute phase error maps of the proposed SAPR-DL method.

The two fringe images generated by DLFT network (one unit-frequency fringe image and one high-frequency fringe image) and the captured one low-frequency fringe image were input into the trained DLPR network to obtain the absolute phase map of the object. To describe this process more concisely, we named it the SAPR-DL method. In order to display the performance of SAPR-DL method more intuitively, the calculated absolute phases of the above four scenarios are shown in Figure 5. Figure 5a–d are the captured low-frequency fringe images. The phase calculated by using the 16-step phase-shifting and multi-frequency temporal phase unwrapping algorithm (MF-TPU) as the ground truth phase maps is shown in Figure 5e–h. Figure 5i–l show the absolute phase calculation results of our SAPR-DL method. Figure 5m–p are absolute phase error maps corresponding to the whole measurement area.

Scenarios 1 and 2 are objects with continuous surfaces. It should be noted that they never appeared in the training data set. Figure 5e,f,i,j show the ground truth phase maps and absolute phase of the SAPR-DL method, respectively. It can be seen from the results that the absolute phase calculated by SAPR-DL method was very similar to the corresponding ground truth, making it difficult to distinguish the difference between them with the naked eye. To show the difference more intuitively, Figure 5m,n show the phase error maps of scenarios 1 and 2. As can be seen from the distribution of the phase error maps, the error was mainly concentrated at the edge of the object. We also tested the ability of the SAPR-DL method by reconstructing discontinuous objects. As shown in scenarios 3 and 4, two geometric objects were placed together to form scenarios with isolated surface objects. Figure 5g,h,k,l show the ground truth phase maps and absolute phase of the SAPR-DL method, respectively. It can be seen from the absolute phase error distribution maps in Figure 5o,p that the absolute phase was correctly calculated, and only slight phase error emerged at the edge of the object. In order to further quantify the phase error, we calculated the mean absolute error (MAE) of the absolute phase of the above four scenarios, as shown in Table 2. The MAE errors of the phase error of scenarios 1, 2, 3 and 4 were 0.11 rad, 0.08 rad, 0.15 rad and 0.13 rad, respectively. It can be seen from Table 2 that the proposed SAPR-DL method was capable of retrieving the accurate absolute phase of discontinuous objects with only one low-frequency fringe image.

**Figure 6:**
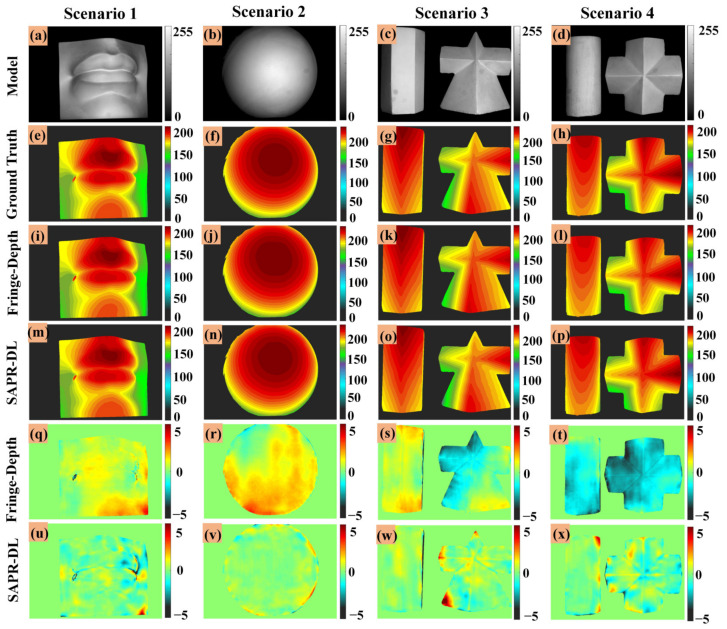
3D reconstruction results based on the proposed SAPR-DL method and Fringe-Depth method. (**a**–**d**) Reconstructed objects. (**e**–**h**) Depth maps (ground truths). (**i**–**l**) Depth maps (Fringe-Depth method). (**m**–**p**) Depth maps (SAPR-DL method). (**q**–**t**) Depth error maps (Fringe-Depth method). (**u**–**x**) Depth error maps (SAPR-DL method).

To show the measurement results of our method more intuitively, we calibrated the projector–camera system using a stereo vision model [37]. Then, 3D shape reconstruction was completed by using absolute phase combined with system calibration parameters. We mapped the point cloud to the z coordinate axis to obtain its depth map. To illustrate the advantages of our SAPR-DL method, we compared our SAPR-DL method with the Fringe-Depth method [24] proposed by Nguyen et al., who used an end-to-end depth neural network to input a single frequency fringe pattern and directly output corresponding depth map. The measurement results of the four scenarios are shown in Figure 6. Figure 6a–d are objects to be measured. Figure 6e–p are ground truth depth maps, the depth maps of the Fringe-Depth method, and the depth maps of the proposed SAPR-DL method, respectively. To clearly display the results, the depth maps of each scenario and corresponding ground truth were displayed in pseudo color, and different depth values were determined by color. In order to evaluate the difference between the reconstructed depth maps and the corresponding ground truth depth maps, we calculated the depth error distribution maps of four scenarios, respectively. Figure 6q–x show the depth error distribution of the Fringe-Depth method and the proposed SAPR-DL method, respectively. It can be seen from Figure 6 that the precision of the Fringe-Depth method was poor and that single frequency fringe image was not able to cope with phase ambiguity/depth ambiguity. The depth error of our SAPR-DL method was far less than that of the Fringe-Depth method, and it can be seen from the error distribution map that the error of our SAPR-DL method mainly occurred at the edge of the object. Notably, Scenario 1 in Figure 6, the flaw in the corner of the mouth is due to the calculation error of the dark fringe area caused by the shooting angle when the traditional algorithm is used to calculate the ground truth. This cannot be completely avoided when calculating ground truth by real experimental data [38].

**Figure 7:**
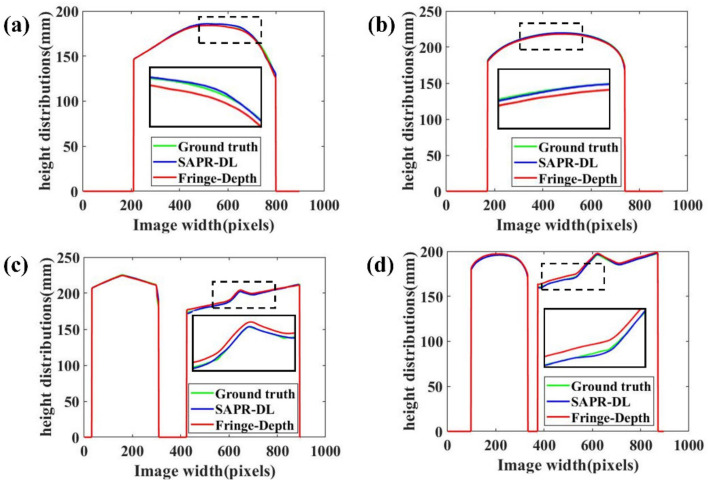
Depth distributions. (**a**) The depth distributions of the 590th row for scenario 1. (**b**) The depth distributions of the 450th row for scenario 2. (**c**) The depth distributions of the 159th row for scenario 3. (**d**) The depth distributions of the 251st row for scenario 4.

Table 2 lists the depth map RMSE of the proposed SAPR-DL method and the Fringe-Depth method. As far as SAPR-DL method is concerned, the RMSE of depth maps of scenarios 1 to 4 are 0.77, 0.43, 0.88 and 0.78, respectively. As far as the Fringe-Depth method is concerned, the RMSE of the depth maps of scenarios 1 to 4 were 1.47, 1.02, 0.95 and 1.42, respectively. Figure 7 shows the depth distribution of an arbitrary line in the four scenarios. The depth distributions of the 590th row in scenario 1, the 450th row in scenario 2, the 159th row in scenario 3 and the 251st row in scenario 4 are plotted in Figure 7a–d. The green, blue and red lines, respectively, represent the depth distribution curve of the ground truth, the proposed SAPR-DL method and the Fringe-Depth method. The black solid line box in Figure 7 is an enlarged display of black dotted line box. From the results in Figure 7, we can see that blue and green lines overlap very well, and the red line has a large offset. Compared with the Fringe-Depth method, the proposed SAPR-DL method exhibited higher reconstruction accuracy. The experimental results suggested that the proposed SAPR-DL method could reconstruct precise 3D shape with only one fringe image even in the case of discontinuous object.

## 4. Discussions

### 4.1. Acquisition of Training Data

The SAPR-DL method we proposed adopts a supervised learning training, which requires a large number of labeled data for training. Although the simulation is one of the important means of making training data sets, the simulation data often deviate from the real data and are subject to limited preset parameters, so it is not completely equal to the real data. Using simulated data sets to train the network will lead to a sharp decline in the performance of the network when faced with real experimental data. In order to make our SAPR-DL method more robust in practical applications, we create a “quasi-experimental” data set by collecting experimental raw data and then use traditional state-of-the-art algorithms to obtain the corresponding ground truth. Unfortunately, the ground truth obtained through experimental data inevitably has errors. Theoretically speaking, the accuracy of our SAPR-DL method can only be infinitely close to the traditional algorithm used for ground truth production. In future research, on the one hand, we can further improve the accuracy of traditional algorithms and obtain higher-quality ground truth; on the other hand, we can design better network models to reduce the error between our result and ground truth.

### 4.2. Visualization of Feature Maps

**Figure 8:**
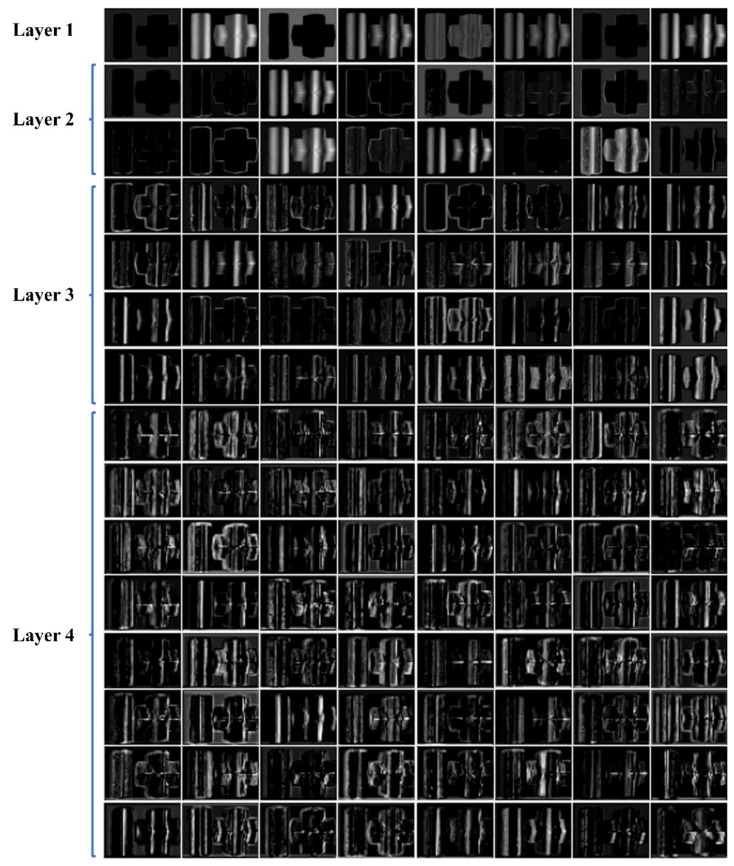
Feature maps of different coding layers based DLFT network.

To interpret the proposed DLFT network as a deeper level, we visualized the feature maps abstracted from the first four layers of the coding path of the DLFT network. The visualization result of Scenario 4 is shown in Figure 8. As is shown in Figure 8, the features of the first and second layers focus mainly on the background and boundary of the target area. The features of the third and fourth layers focus mainly on the distribution of fringe information. The experimental results show that the proposed model separates the background and target region well and makes a good response to the distribution of fringe information.

### 4.3. The Influence of the Number of Fringe Images on the Measurement Results

**Figure 9:**
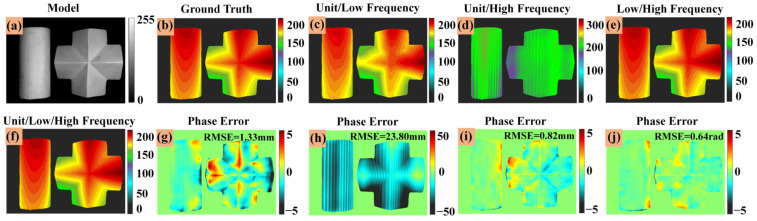
3D reconstruction results of different number of fringe images. (**a**) Reconstructed object (scenario 1). (**b**) Depth map (ground truth). (**c**) Depth map (unit/low frequency). (**d**) Depth map (unit/high frequency). (**e**) Depth map (low/high frequency). (**f**) Depth map (unit/low/high frequency). (**g**) Depth error map (unit/low frequency). (**h**) Depth error map (unit/high frequency). (**i**) Depth error map ((low/high frequency). (**j**) Depth error map (unit/low/high frequency).

In this section we explain why it is necessary to use three fringe images with different frequencies as input to the DLPR model. From the perspective of traditional optical phase calculation, it is more stable and reliable to use as many fringe patterns as possible for phase calculation. In order to verify the influence of different numbers of fringe patterns on the accuracy of phase calculation, we discussed four different cases: unit/low frequency, unit/high frequency, low/high frequency and unit/low/high frequency, respectively. Figure 9a is a reconstructed object. Figure 9b is the depth map (ground truth), and Figure 9c–f are the corresponding depth maps of unit/low frequency, unit/high frequency, low/high frequency and unit/low/high frequency, respectively. Figure 9g–j are the depth error maps of unit/low frequency, unit/high frequency, low/high frequency and unit/low/high frequency, and the RMSE of depth maps are 1.33, 23.80, 0.82 and 0.64, respectively. It can be seen from the figure that the result of using three frequency fringe images is the best performance because it provides more fringe information.

### 4.4. Experimental Results Comparison of Different Network Architectures

**Figure 10:**
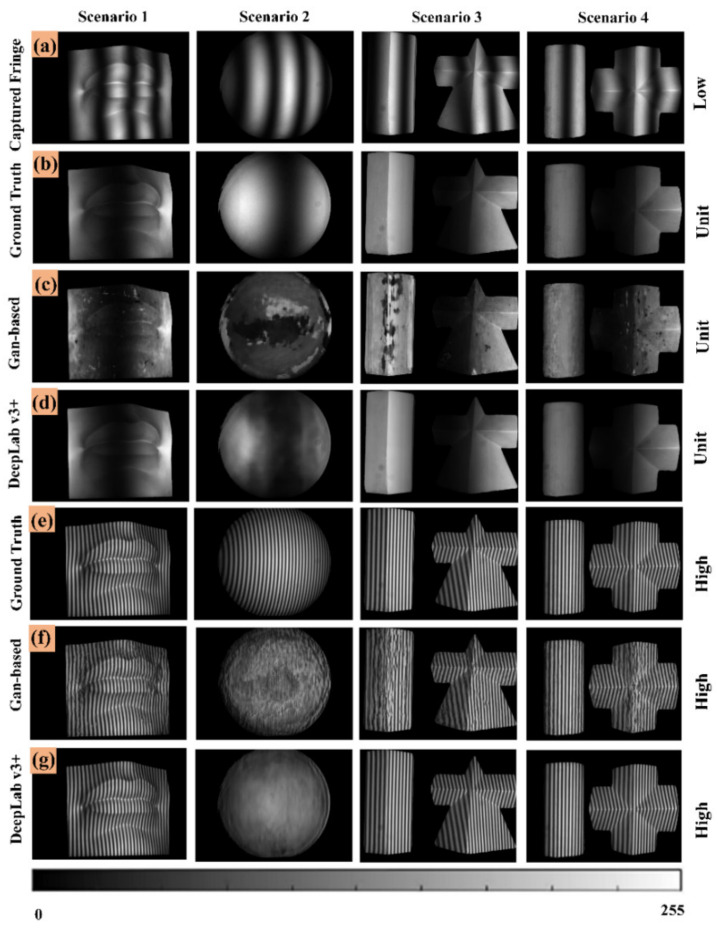
Fringe images generated by different network architectures. (**a**) Captured low-frequency fringe images. (**b**) Ground truths (unit-frequency). (**c**) Unit-frequency fringe images generated by the Gan-based network. (**d**) Unit-frequency fringe images generated by the DeepLabv3+ network. (**e**) Ground truths (high-frequency). (**f**) High-frequency fringe images generated by the Gan-based network. (**g**) High-frequency fringe images generated by the DeepLabv3+ network.

To explore the performance of different network structures in our proposed method, we compared the experimental results of the DeepLabv3+ structure and the Gan-based structure. The corresponding experimental results are shown in Figure 10. Figure 10a is captured low-frequency fringe images. Figure 10b is ground truths of unit-frequency fringe images. Figure 10c is unit-frequency fringe images generated by the Gan-based network. Figure 10d is unit-frequency fringe images generated by the DeepLabv3+ network. Figure 10e is ground truths of high-frequency fringe images. Figure 10f is high-frequency fringe images generated by the Gan-based network. Figure 10g is high-frequency fringe images generated by the DeepLabv3+ network. According to the SSIM index in Table 3, Gan-based performance is the worst, and the SSIM index of high-frequency fringe image in Scenario 2 is only 0.525. As far as the DeepLabv3+ structure is concerned, the SSIM indexes of Scenarios 1, 3 and 4 are above 0.951; the SSIM indexes of the generated low frequency and high frequency fringe images are 0.881 and 0.551 in Scenario 2, respectively. The experimental results show that DeepLabv3+ structure is not stable enough in the generation of fringe images.

## 5. Conclusions

In summary, a single-shot multi-frequency absolute phase retrieval method based on deep learning (SAPR-DL) was proposed in this paper, which can retrieve the absolute 3D information of complex scenarios with large surface discontinuities or isolated objects under the condition of projecting single-shot fringe pattern. By inputting the captured one low-frequency fringe image into the DLFT network, the other two fringe images (unit-frequency and high-frequency) required to determine the reliable absolute phase can be transformed. The absolute phase can be obtained by inputting three fringe images with different frequencies into the trained DLPR network. The experimental results show that our SAPR-DL method only needs to collect one fringe image and can precisely measure the 3D shape of discontinuous or isolated objects, providing a new idea for rapid 3D shape measurement.

## Figures and Tables

**Table 1:** Structure similarity indexes of fringe images generation with different frequencies. U represents the unit-frequency fringe; L represents the low-frequency fringe; H represents the high-frequency fringe.

SSIM	U (input)		L (Input)		H (Input)	
	L	H	U	H	U	L
Scenario 1	0.984	0.970	0.979	0.982	0.979	0.985
Scenario 2	0.987	0.974	0.952	0.989	0.950	0.988
Scenario 3	0.976	0.932	0.972	0.973	0.971	0.975
Scenario 4	0.972	0.970	0.984	0.984	0.983	0.972

**Table 2:** Quantitative comparison of the proposed SAPR-DL method and Fringe-Depth method in terms of RMSE of depth map.

RMSE (mm)	Scenario 1	Scenario 2	Scenario 3	Scenario 4
SAPR-DL	0.77	0.43	0.88	0.78
Fringe-Depth	1.47	1.02	0.95	1.42

**Table 3:** Comparison of different network architectures.

SSIM (L/H)	Scenario 1	Scenario 2	Scenario 3	Scenario 4
Gan-based	0.923/0.907	0.706/0.525	0.850/0.782	0.911/0.878
DeepLabv3+	0.973/0.970	0.881/0.551	0.962/0.951	0.978/0.970
DLFT	0.979/0.982	0.952/0.989	0.972/0.973	0.984/0.984
